# Choroidal Metastasis With Secondary Retinal Detachment in a Man With BCRA2-Associated Breast Adenocarcinoma

**DOI:** 10.7759/cureus.24243

**Published:** 2022-04-18

**Authors:** Nedda Sanayei, Samaneh Davoudi, Alexander Port, Xuejing Chen

**Affiliations:** 1 Ophthalmology, Boston Medical Center, Boston, USA

**Keywords:** b-scan, retinal detachment, choroidal metastasis, breast cancer, brca2

## Abstract

Breast cancer with ocular metastasis is rare in men. Such cases with BRCA2 gene mutation are even less common. This case report describes a 59-year-old man with recently diagnosed breast adenocarcinoma who presented for surgical repair of a retinal detachment in his left eye. Clinical exanimation revealed a left inferior retinal detachment without a clear retinal break. Spectral-domain optical coherence tomography (SD-OCT) of the left macula showed sub-retinal fluid extending into the fovea and a thickened choroid. B-scan ultrasonography was obtained and showed a diffuse inferior choroid mass measuring 5 millimeters (mm) at the apex with an overlying retinal detachment, consistent with breast cancer metastasis to the choroid. He was initiated on chemotherapy and whole-brain radiation with symptomatic improvement. This case illustrates the importance of obtaining a thorough medical history and additional testing when indicated.

## Introduction

Breast cancer in men accounts for only 1% of cases of all breast cancer cases in the United States [1]. The majority of male breast cancer cases occur spontaneously without known associated risk factors; however, when a genetic association exists, a mutation of the BRCA2 gene is most common. We report a rare case of a man who presented with a serous retinal detachment and a choroidal lesion that was found to be metastasis from BRCA2-associated breast cancer.

## Case presentation

**Figure 1:**
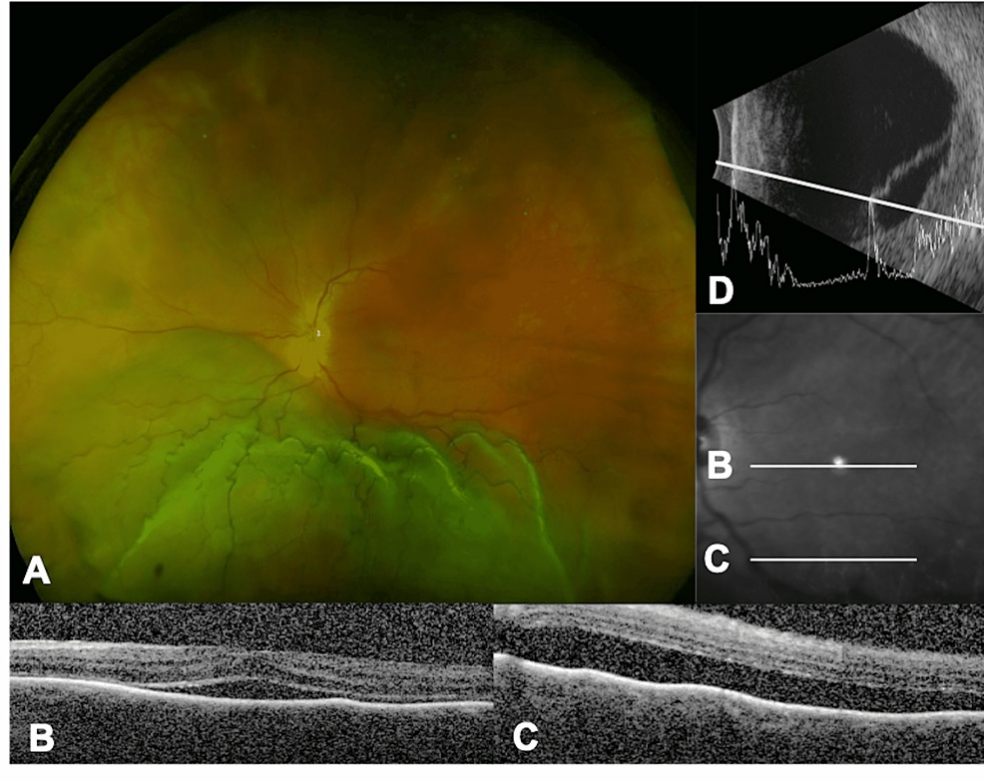
Fundus photo, Spectral domain-OCT and B-scan of left eye Examination at presentation showed an inferior retinal detachment of the left eye with no clear retinal breaks or masses (A). Spectral domain-OCT revealed an inferior retinal detachment of the left eye with a thickened choroid and sub-retinal fluid involving the fovea (B) and extending inferiorly with the lump bumpy appearance (C). B-scan ultrasonography confirmed a diffuse inferior choroidal mass measuring 5 mm at the apex with overlying retinal detachment (D). OCT: optical coherence tomography

**Figure 2:**
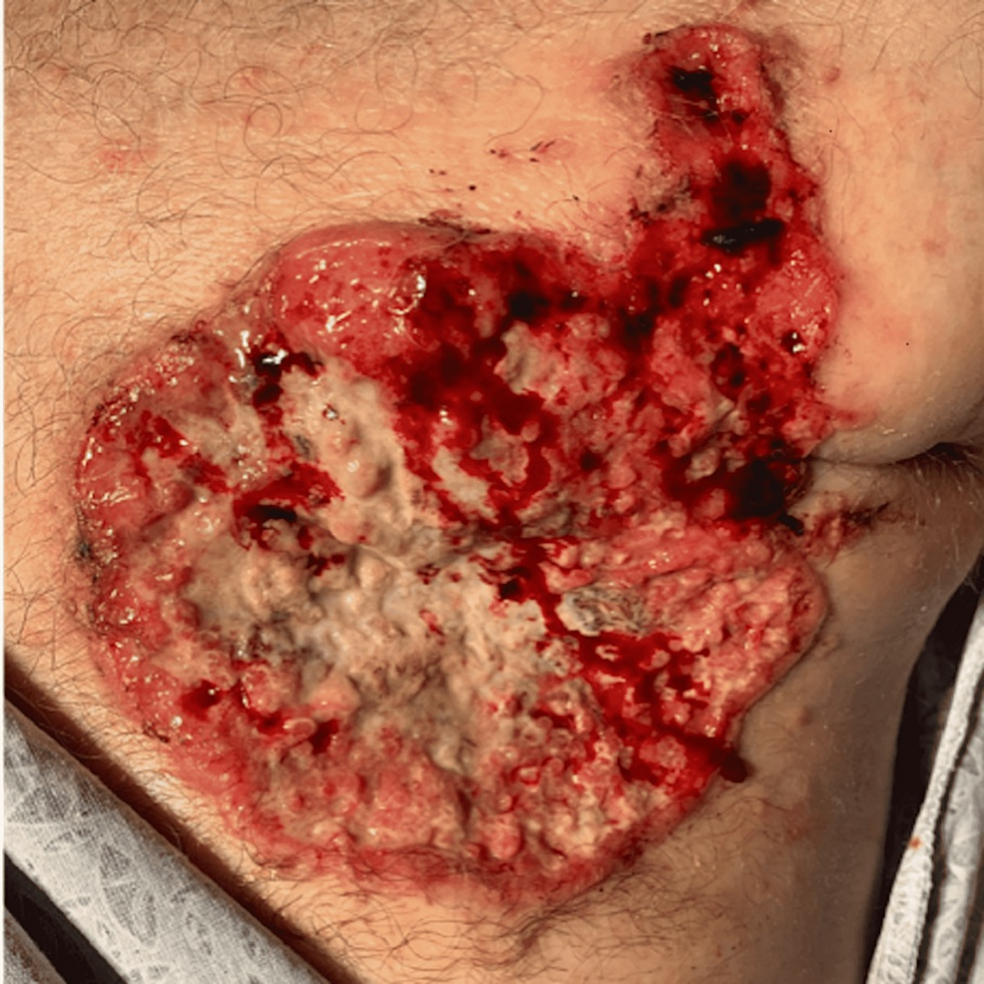
Ulcerating left breast mass Ulcerating left breast mass found on initial presentation to an emergency department two weeks before presentation to ophthalmology. Superficial punch biopsy revealed ER+/PR+/HER2+ breast adenocarcinoma. ER = estrogen receptor, PR = progesterone receptor, HER2 = human epidermal growth factor receptor 2

A 59-year-old White man was referred for immediate surgical repair of a painless retinal detachment in his left eye. The patient reported six weeks of photopsia and loss of the left temporal visual field. Ocular history was pertinent for moderate myopia (-2.75 diopters in the right eye and -2.00 diopters in the left eye). Visual acuity was 20/20 in the right eye and 20/100 in the left eye. The confrontational visual field was full in the right eye with total superior temporal and superior nasal deficiencies in the left eye. Anterior segment examination was normal in both eyes. Dilated fundus examination of the left eye was notable for a bullous inferior retinal detachment (Figure 1A). There were no clear tears or masses detected on a depressed exam. Spectral-domain optical coherence tomography (SD-OCT) of the left macula showed sub-retinal fluid extending into the fovea with a thickened choroid and the lumpy bumpy appearance (Figure 1B, 1C). B-scan ultrasonography was obtained and showed a diffuse inferior choroidal mass measuring 5 mm at the apex beneath the retinal detachment (Figure 1D), Upon further discussion with the patient, he revealed that over the past eight months, he has had a progressively enlarging ulceration of the left anterior chest wall and underwent a biopsy given suspicion for neoplasm in the emergency room two weeks ago (Figure 2). Additional family history revealed a sister with a BRCA2 gene mutation and ovarian cancer, as well as a father with pancreatic cancer. Surgical repair was canceled, and the patient was referred to oncology.

**Figure 3:**
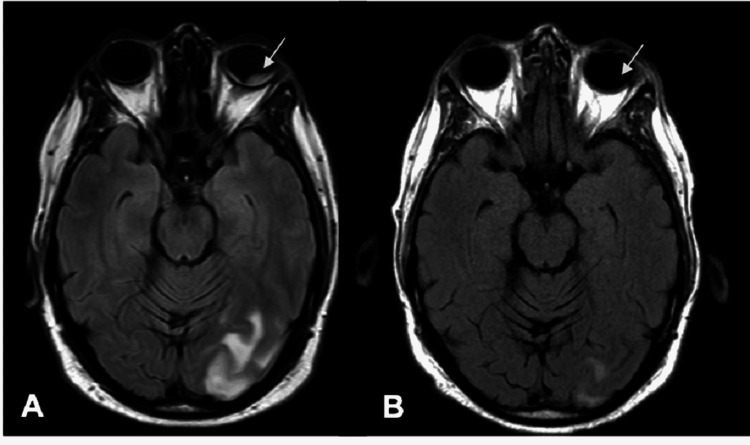
T2/FLAIR brain magnetic resonance imaging Axial images reveal a hyperintense lesion of the left posterior pole of the left globe (arrow-head) as well as left occipital lobe enhancement (A). Repeat imaging three months later shows the resolution of the left orbital mass and interval decrease in brain lesions following treatment (B). T2/FLAIR: T2-weighted fluid-attenuated inversion recovery

Further characterization of the recently biopsied breast mass showed estrogen receptor-positive, progesterone receptor-positive, human epidermal growth factor receptor-positive (ER+/PR+/HER2+) BRCA2 gene mutation-associated breast cancer. Subsequent computed tomography of the chest, abdomen, and pelvis revealed metastases to the lung, skin, lymph nodes, and right adrenal gland. T2-weighted fluid-attenuated inversion recovery (T2/FLAIR) MRI of the brain following ophthalmic evaluation revealed multiple enhancing lesions in both cerebral and cerebellar hemispheres with surrounding edema. Additionally, there was an enhancement of the left orbit most prominent inferiorly, consistent with metastasis to the choroid and the previously performed B-scan (Figure 3A). The patient was initiated on chemotherapy with docetaxel, pertuzumab, and trastuzumab as well as whole-brain radiation. Repeat MRI of the brain following whole brain radiation and five cycles of chemotherapy showed radiographic resolution of the left orbital mass and significant improvement in the size of the intracranial enhancing lesions (Figure 3B). The patient reported symptomatic improvement of vision and resolution of the photopsia.

## Discussion

The choroid is the most common site for intraocular metastases with its rich vascular supply. Shields et al. published a retrospective study of 2214 uveal metastases diagnosed between 1974 and 2017 at Wills Eye Hospital where metastases to the choroid accounted for 90% of all uveal metastases [2]. In women, the most common primary tumor site was the breast (58%) while in men the lung was found to be the most common (40%). Breast cancer in men accounts for less than 1% of all breast cancer diagnoses in the United States and is consistent with the rate of choroidal breast cancer metastases in women versus men published by Shields et al. [1,2] when they found 413 female uveal tumors versus only three male uveal tumors originating from the breast [2]. Assuming a similar rate of metastasis to the choroid as in women, Kreusel et al. estimate that there are only 25 cases of male breast cancer with metastases to the choroid per year [3].

BRCA1 and BRCA2 are tumor suppressor genes located on chromosome 17q21 and 13q12.13, respectively, and are transmitted with an autosomal dominant inheritance pattern. Mutations in both of these genes, especially BRCA2, have been associated with an increased risk of breast cancer in men. A retrospective study of 1948 families with 97 men with breast cancer found that 26% of the subjects had mutations of the BRCA2 gene. They estimated the cumulative risk for breast cancer in men with a BRCA2 mutation to be 6.8% which is about 100 times higher than the general male population [4]. Given this correlation, men diagnosed with breast cancer should be referred for genetic counseling and careful family oncologic history should be obtained. Other risk factors of breast cancer in males are aging, positive family history, Klinefelter syndrome, Kartagener syndrome, radiation exposure to chest, liver disease, estrogen treatment, alcohol, obesity, and testicular conditions (such as undescended testicle, having mumps as an adult, history of orchiectomy).

Our case presents a rare report of a man with BRCA2-associated breast cancer that presented with a choroidal metastasis and secondary serous retinal detachment. Although the BRCA2 gene mutation significantly increases the risk of breast cancer in men, the combination of relatively infrequent, and likely under-reported, male breast cancer cases along with choroidal metastases and the presence of BRCA2 gene mutation makes for an uncommon combination.

The use of B-scan ultrasonography served a crucial role in this case and often is essential in the diagnosis and characterization of choroidal tumors. It allows for indirect visualization and characterization of the mass as well as any overlying retinal detachments. Typically, diagnosis of choroidal metastasis can be made clinically with fundus examination and ancillary imaging. However, fine-needle aspiration biopsy (FNAB) can also be used to verify the diagnosis in cases that cannot be confirmed through less invasive techniques. According to Shields et al., of 159 patients obtaining FNAB of suspected intraocular tumors, the most common complication was intraocular hemorrhage (13%) without subsequent retinal detachments or tumor recurrence [5].

Given that these patients with choroidal metastases by definition have stage IV disease, treatment is generally considered palliative with an average five-year relative survival rate of 27% for individuals with metastatic breast cancer [6]. Treatment must thus be individualized to the extent of the systemic involvement with the goal of symptomatic management and prolongation of life. Common treatment modalities involve systemic chemotherapy and hormonal treatment in hormone receptor-positive cases like our patient. Localized ocular treatment options include external beam radiotherapy and plaque radiotherapy [7]. External beam radiotherapy is well-established and can be used in cases that fail systemic therapy alone. Plaque brachytherapy allows for radiation delivery directly to the choroidal lesion and thus has a role in isolated solitary lesions. Side effects can include radiation retinopathy, dry eyes, and cataract formation. Manquez et al. found choroidal metastases regression with aromatase inhibitors (anastrozole, letrozole, and exemestane) in 10 out of 17 patients with hormone receptor-positive breast cancer over a mean follow-up of 20 months [8]. Our patient’s treatment plan included systemic chemotherapy with docetaxel, pertuzumab, and trastuzumab which are targeted to HER-2 positive breast cancers. Systemic chemotherapy is typically the first line in combination with whole-brain radiation in the setting of central nervous system involvement such as with our patient. Our patient subjectively reports an improvement of symptoms since initiation of treatment which corresponds to the radiographic resolution of the orbital mass on repeat imaging.

## Conclusions

This is a rare case of a patient who was referred urgently to the retina clinic for surgical repair of a unilateral exudative retinal detachment. Careful examination, imaging, and medical history revealed that the retinal detachment was secondary to a choroidal metastasis. First-line treatment, in this case, is the systemic treatment for his cancer as opposed to surgical repair. This report illustrates the importance of careful history taking and considering B-scan ultrasonography in ambiguous cases of retinal detachments without a visible break.

## References

[1] Siegel RL, Miller KD, Jemal A (2019). Cancer statistics, 2019. CA Cancer J Clin.

[2] Shields CL, Welch RJ, Malik K (2018). Uveal metastasis: clinical features and survival outcome of 2214 tumors in 1111 patients based on primary tumor origin. Middle East Afr J Ophthalmol.

[3] Kreusel KM, Heimann H, Wiegel T, Bornfeld N, Foerster M (1999). Choroidal metastasis in men with metastatic breast cancer. Am J Ophthalmol.

[4] Tai YC, Domchek S, Parmigiani G, Chen S (2007). Breast cancer risk among male BRCA1 and BRCA2 mutation carriers. J Natl Cancer Inst.

[5] Shields JA, Shields CL, Ehya H, Eagle R, Potter P (1993). Fine-needle aspiration biopsy of suspected intraocular tumors. The 1992 Urwick Lecture. Ophthalmology.

[6] Breast Cancer Facts & Figures. https://www.cancer.org/research/cancer-facts-statistics/breast-cancer-facts-figures.html..

[7] Chen CJ, McCoy AN, Brahmer J, Handa JT (2011). Emerging treatments for choroidal metastases. Surv Ophthalmol.

[8] Manquez ME, Brown MM, Shields CL, Shields JA (2006). Management of choroidal metastases from breast carcinomas using aromatase inhibitors. Curr Opin Ophthalmol.

